# The Effects of Titanium Surfaces Modified with an Antimicrobial Peptide GL13K by Silanization on Polarization, Anti-Inflammatory, and Proinflammatory Properties of Macrophages

**DOI:** 10.1155/2020/2327034

**Published:** 2020-07-24

**Authors:** Xuxi Chen, Lin Zhou, Dong Wu, Wenxiu Huang, Yanjun Lin, Bowei Zhou, Jiang Chen

**Affiliations:** ^1^Fujian Key Laboratory of Oral Diseases, School and Hospital of Stomatology, Fujian Medical University, China; ^2^Fujian Provincial Engineering Research Center of Oral Biomaterial, School and Hospital of Stomatology, Fujian Medical University, China; ^3^Stomatological Key Lab of Fujian College and University, School and Hospital of Stomatology, Fujian Medical University, China; ^4^Research Center of Dental and Craniofacial Implant, School and Hospital of Stomatology, Fujian Medical University, China; ^5^Institute of Stomatology, School and Hospital of Stomatology, Fujian Medical University, China; ^6^Department of Oral Implantology, School and Hospital of Stomatology, Fujian Medical University, China; ^7^Institute of Stomatology & Research Center of Dental and Craniofacial Implant, School and Hospital of Stomatology, Fujian Medical University, China

## Abstract

The polarization of macrophages and its anti-inflammatory and proinflammatory properties play a significant role in host response after implant placement to determine the outcome of osseointegration and long-term survival. In the previous study, we immobilized an antimicrobial peptide, GL13K, onto titanium surfaces to provide immune regulation property. In the herein presented study, we aimed at investigating whether GL13K immobilized titanium surface could improve osteogenesis and reduce the inflammatory reaction around the biomaterials by altering macrophage response. We evaluated the cell proliferation of the different phenotypes of macrophages seeded in GL13K-coated titanium surface, which indicated an inhibition of M1 macrophages and a good cytocompatibility to M2 macrophages. Then, we measured the inflammatory and anti-inflammatory activity of the M1 and M2 macrophages seeded on the GL13K-coated titanium surfaces. The results of the enzyme-linked immunosorbent assay and quantitative reverse transcription-polymerase chain reaction showed that the group with the GL13K modified surface had a downregulation in the expression level of the tumor necrosis factor-*α* and interleukin-1*β* in M1 macrophages and an upregulation of IL-10 and transforming growth factor-*β*3 (TGF-*β*3) levels in M2 macrophages. This study demonstrated that the GL13K modified titanium surfaces can regulate macrophages' polarization and the expression of inflammatory and anti-inflammatory effects, reducing the effects of the inflammatory process, which may promote the process of bone regeneration and osseointegration.

## 1. Introduction

Based on the developing of dental implant technology and biomaterials, dental implant has become an increasingly popular treatment for missing teeth. The success of the osseointegration and the subsequent implant survival depend from multiple causal factors, such as physiological conditions of the recipient, implant site preparation, implant design, and implant surface properties. Dental implant is a prosthetic device made of alloplastic material such as titanium and its alloys like Ti-6Al-4V for its excellent physical and chemical properties and biocompatibility. On the other hand, furthermore factors such as nature of the implant surface and implant placement procedure also contribute to the final success of osseointegration. However, both the implant operation as a traumatic operation and the implant as a foreign body inevitably lead to a significant immune response and the consequent biological behavior of bone cells, which finally determine the fate of the dental implant. Therefore, the immune response may become an important factor that has a potentially effect on the osteogenic capability of bone biomaterial. However, the current research on implants and bone biomaterials mainly focuses on promoting implant osseointegration and inducing osteogenesis, but ignores the important role of immune-inflammatory response in this process, which often leads to conflicts and disagreements between in vivo and in vitro studies. In order to improve this situation, many new researches began to try to incorporate immune cells into the in vitro evaluation system for osteogenic performance of implants and bone biomaterials in recent years, and good progress was made [1–4]. In vivo and in vitro matching results are also helpful for the research and development of implant and bone biomaterials.

The immune system plays an important role in the host response after implant placement to determine the outcome of osseointegration and long-term survival. As part of the immune system, macrophages receive the most attention due to their vital roles in the regulation of inflammation and tissue regeneration. Studies have shown the high plasticity and multiple effects of macrophages during the healing phase [5]. At the inflammatory site, macrophages activated by infectious microorganism-related molecules and inflammation-related cytokines can switch into different phenotypes, secrete many cytokines, and create a different immune environment [6]. In a specific immune microenvironment, macrophages can polarize into M1/M2 phenotypes characterized by their different functions, surface markers, and inducers that mirror the Th1/Th2 nomenclature of T helper cells [7]. M1 macrophages, known as classically activated inflammatory phenotype, express high levels of the cytokine interleukin- (IL-) 12 as well as the cytokine IL-23. On the contrary, the expression level of the cytokine IL-10 is low in the M1 macrophages. They also secrete many kinds of proinflammatory cytokines including tumor necrosis factor-*α* (TNF-*α*), IL-6, and IL-1*β*, and produce toxic effector molecules, like reactive oxygen species (ROS), and nitric oxide (NO) [8]. Alternative activated M2 macrophages are characterized by having a high level of scavenger-, mannose, and galactose-type receptors and expressing a high level of IL-10 and low level of IL-12 and IL-23 [9].

The development of osteoimmunology has revealed the multiple functions of macrophages in the bone regeneration process. For example, M1 macrophages secrete many cytokines (TNF-*α*, IL-6, IL-1*β*), which are generally recognized to be inflammatory and have properties of inducing osteoclastogenesis and leading to bone resorption. However, some recent researches have demonstrated the enhancement of osteogenesis in the response of M1 macrophages, rather than M2 [10], while in a wound healing environment, M2 macrophages seem to be related to the late stage of tissue healing. They secrete not only osteogenic cytokines which contributed to osteogenesis, such as (BMP2) and (VEGF), but complicated inflammatory and fibrous agents as well, like TGF-*β*, which lead to inflammation and forming of fibrous capsules [11]. It seems that M2 macrophages play a more important role in the repair reaction compared with M1 macrophages, while the M1 macrophages can determine the pattern of cytokines secreted by M2 macrophages during the early phase of bone regeneration. Prolonged M1 polarization results in the release of fibrosis-related cytokines. Conversely, an effective and timely switch in M1 polarization can lead to osteogenesis-enhancing cytokines release pattern of M2 macrophages [12]. Accordingly, it is probable that both macrophage phenotypes play indispensable roles during the bone regeneration process, and that the switch pattern of macrophage determines the fate of bone biomaterials. Therefore, we can modulate the response of macrophages to biomaterials and affect the bone formation process. An advanced generation of the biomaterials for implants ought to have a property of regulating the local immune environment to improve osseointegration and osteogenesis around the implant.

Antimicrobial peptides (AMPs) have received extensive attention in the area of biomaterials in recent years because of their broad-spectrum antibacterial activity, reduced cytotoxicity, nonselection of resistant mutants, and anti-inflammatory activity, especially when immobilized onto a titanium surface [13, 14]. Our previous research about titanium surface immobilized with antimicrobial peptide GL13K using silane as a chemical linker demonstrated its good antibacterial activity against Porphyromonas gingivalis and Staphylococcus epidermidis with no cytotoxicity to human gingival fibroblasts and osteoblasts [15, 16]. The GL13K immobilized titanium surface has a property of reducing the effects of the inflammatory process through the downregulation of the main proinflammatory cytokine expression and upregulation of the anti-inflammatory cytokine expression without any influence on cell attachment and proliferation [17]. The immunomodulation property of the titanium surface immobilized with AMP GL13K to macrophage polarization still requires clarification.

According to our previous works, we immobilized an antimicrobial peptide, GL13K, onto titanium surfaces to prepare a potential bone biomaterial and observed a porous network on the GL13K coated surface with greater roughness nanotopography. This study aimed at exploring the impact of the surface-immobilized GL13K to the macrophage polarization and analyzing the proliferation and secretion of different macrophage phenotypes on the titanium surface immobilized with GL13K so as to explain the immunomodulation property of this biomaterial surface in the host inflammatory process and the macrophage polarization. This research provides rationales for the osteoimmunomodulation and osteogenesis promotion of the new generation of implant biomaterials.

## 2. Materials and Methods

### 2.1. Titanium and the Modification of the Titanium Surfaces with the AMP GL13K

We used pure titanium foils supplied by Alfa Aesar as the sample in control groups. And the antimicrobial peptides (GL13KGKIIKLKASLKLLCONH2, MW = 1424 g/mol) which were used to immobilize the titanium surfaces were provided by China peptides Co. Ltd. (Shanghai, China). The experimental groups were made by modifying the same pure titanium foils with antimicrobial peptide GL13K. We use 3-(chloropropyl)-triethoxysilane (CPTES, 95%) which was supplied by Sigma-Aldrich (St. Louis, MO, USA) as a linker to modify the titanium surface with the antimicrobial peptide GL13K, as mentioned in our previous study [15]. In simple terms, the immobilization can be immobilized while treated by NaOH and dipped in a mixture, which contained 0.6 ml of diisopropylethylamine, 1.2 ml of 3-(chloropropyl)-triethoxysilane, and 7 ml of anhydrous pentane for silanization. Then, silanized titanium foils obtained were used in Sil-Ti groups. After that, we modified the titanium surface with the antimicrobial peptide GL13K by dipping in a mixed solution which contained AMP GL13K and NA_2_CO_3_ overnight. Ethanol was used to disinfect all samples for 2 h. Then, the material surfaces were washed with deionized water and dried before using in the GL13K-Ti groups in further assays. We set 0.1 mM as the appropriate saturation concentration of AMP GL13K as we suggested in our previous research [17]. Five samples which were both 10 × 10 mm square, and 0.25 mm thick were used in each group for this study.

### 2.2. Cell Culture

RAW 264.7 cells, a murine leukemic monocyte cell line obtained from the American Type Culture Collection (ATCC), were used in this study. RAW 264.7 cells were incubated at 37°C under a humidified a 5% CO_2_ atmosphere with high glucose Dulbecco's Modified Eagle's Medium (DMEM) (Hyclone, USA) supplemented with 1% penicillin/streptomycin (100 ×, Beyotime, China) and 10% fetal bovine serum (Hyclone, USA). RAW 264.7 cells were passaged by gently scraping the cells off while the RAW 264.7 cells reached the confluence of approximately 70%. ALL the RAW 264.7 cells used for the assays were from third to fifth passages.

### 2.3. Macrophage Activation

When activated cells were needed, Raw 264.7 cells were cultured with the stimulation of 100 ng/ml of ultra-pure lipopolysaccharides(LPS, E. coli, Invivogen, USA) after incubating as described previously to prepared M1 macrophages, and M2 macrophages were obtained by receiving the stimulation of 50 ng/ml of recombinant murine IL-4 (E. coli, PeproTech Inc., USA) after early incubation.

### 2.4. Macrophage Polarization

The polarization of the macrophages in different groups was evaluated by flow cytometry. After culturing for 24 h, 72 h, and 7 d, macrophages were collected and transferred into 2 groups. The cells in the first group, classified as M1 identification, were incubated with the CD11c antibody marked by PE-Cy7. The cells in the second group, classified as M2 identification, were incubated with the CD206 antibody marked by Alexa Fluor 647. After 1 h of incubation on ice, the detection was performed on the BD FACSCanto™ II system to analyze the surface marker expression of different macrophage polarizations.

### 2.5. Macrophage Proliferation

The proliferation of the macrophages with different polarizations after culturing on the biomaterial surfaces for 24 h, 48 h, and 72 h was evaluated by the Cell Counting Kit-8 (CCK-8). At each culturing time point, the old medium was replaced with 500 *μ*l of fresh culture medium and 50 *μ*l of CCK-8 solution in each group which were incubated for 2 h at 37°C. The optical absorbance value of the solution was measured by a microplate reader at a wavelength of 450 nm.

### 2.6. Inflammatory and Anti-Inflammatory Cytokines Expression

The inflammatory and anti-inflammatory cytokines expressions of macrophages with different polarizations in different groups were detected by enzyme-linked immunosorbent assay (ELISA). After 24 h culturing on the different surfaces and activated as described previously when cells reached the confluence of 80%, the collected culture medium was centrifuged at 4°C. Extracellular levels of TNF*α*, IL-1*β* of M1 macrophages and IL-10, IL-1ra of M2 macrophages were evaluated by ELISA according to the manufacturer's instructions (ABclonal. USA).

### 2.7. Inflammatory and Anti-Inflammatory Gene Expression

The expression level of inflammation-related and anti-inflammatory genes were measured by quantitative reverse transcription-polymerase chain reaction (qRT-PCR) in the M1 macrophage and M2 macrophage groups, respectively, and the results were normalized to the expression of house-keeping gene glyceraldehyde 3-phosphate dehydrogenase (GAPDH). After the macrophages with different polarizations were seeded onto different surfaces in 6-well plates for 3 days, the total RNA of the treated macrophages with different polarizations was isolated from the different groups following a conventional method. Then, the extracted RNA was transcribed into cDNA by the PrimeScript RT Reagents Kit (Takara) following the manufacturer's instructions after the purity and concentration. The real-time qPCR was performed using the SYBR Premix Ex Taq (Takara) and conducted on a Roche LightCycler 480 System. After the completion of the reaction, the expression level of each gene was calculated by the software of the instrument using the 2^-*ΔΔ*CT^ method. The values were expressed as mean ± standard deviation. The primer sequences and genes studied in this section are all listed in Table 1.

### 2.8. Statistical Analysis

The mean values ± standard deviation was used to express the data of this study. Statistical analysis was carried out with SPSS statistics version (IBM USA) on different samples. All the data were analyzed with a one-way analysis of variance (ANOVA), and the least significant difference (LSD) method was used for comparison. A difference of *p* value < 0.05 was considered as statistically significant.

## 3. Results

### 3.1. Macrophage Polarization

**Figure 1:**
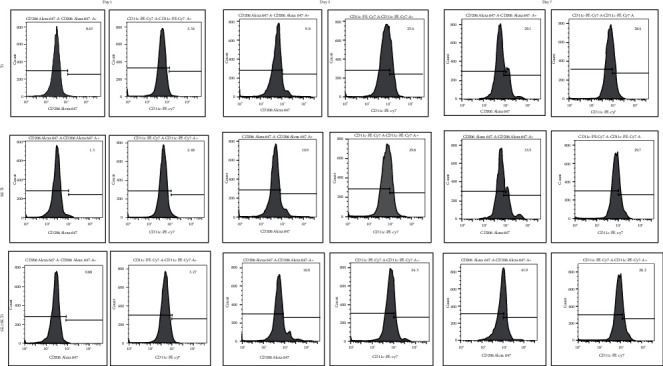
FACS results of RAW264.7 cells cultured on the titanium surface, the silanized titanium surface, and the GL13K-coated surface for 24 h, 72 h, and 7 d. After being seeded onto GL13K-coated titanium surfaces, the mean expression level of CD206 was increased in comparison to that in the titanium groups, while the mean expression level of CD11c was reduced in the cells cultured on the GL13K-coated titanium compared with the titanium group.

After culturing on different surfaces for 24 h, 72 h, and 7 d, the expression of the macrophage surface marker evaluated by flow cytometry showed higher expression level of the M2 marker CD206 and lower expression level of the M1 marker CD11c by the RAW264.7 cells in the GL13K groups in comparison with those in the titanium groups (Figure 1). These results suggested that GL13K-coated titanium surface has a better property of reducing the M1 polarization of macrophages and increasing the M2 polarization of macrophages then the titanium surface.

### 3.2. Macrophage Proliferation

**Figure 2:**
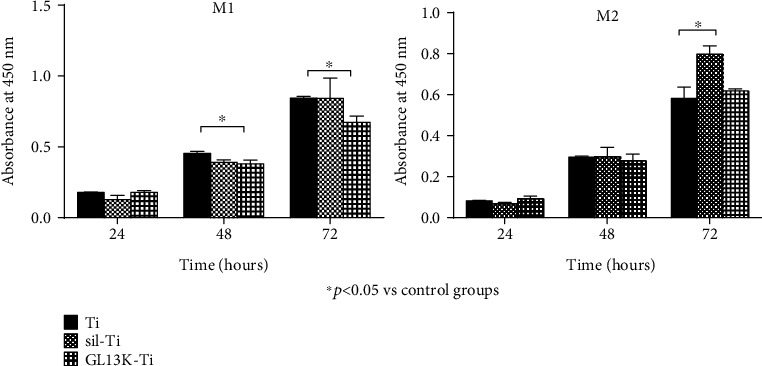
Cell proliferation of M1 macrophages and M2 macrophages after culturing on the titanium surface, the silanized titanium surface, and the GL13K-coated surface for 24, 48, and 72 h. Error bars represent mean ± SD for *n* = 5. A statistically significant difference exists between GL13K-coated Ti groups and Ti groups at 48 h (*p* < 0.001) and 72 h (*p* < 0.05).

The proliferation of macrophages was measured by using CCK-8 in different groups. The results in Figure 2 showed the difference of the optical density (OD) values in different groups. For the macrophages with the M1 polarization, the statistically significant differences can be found between the results on the titanium surface and GL13K-coated titanium surface at both 48 h and 72 h. By contrast, the results for the macrophages with M2 polarization showed no statistically significant difference between the two groups which mean that they were almost identical in the 3 time periods. Taken together, all these results show that GL13K immobilized titanium surface may inhibit the proliferation of M1 macrophages and have a great biocompatibility for M2 macrophages.

### 3.3. Inflammatory and Anti-Inflammatory Cytokine Expression

**Figure 3:**
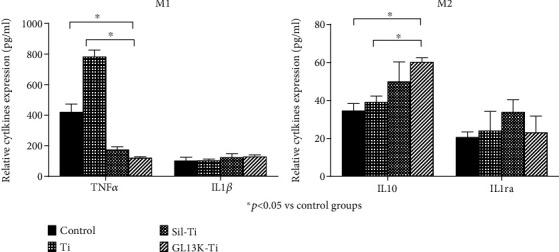
ELISA analysis of the secretion of IL-1*β* and TNF-*α* in macrophages with M1 polarization and IL-10 and IL-1ra in macrophages with M2 polarization cultured on the titanium surface, the silanized titanium surface, and the GL13K-coated surface at 24 h. Error bars represent mean ± SD for *n* = 5. The downregulation of the secretion level of cytokines TNF-*α* (*p* < 0.001) and the upregulation of cytokines IL-10 (*p* = 0.002) were detected in the GL13K immobilized titanium group.

ELISA was used to evaluate the extracellular secretion level of cytokines TNF-*α*, IL-1*β* in macrophages with M1 polarization, and the cytokines IL-10 and IL-1ra in macrophages with M2 polarization. While comparing with the macrophages in the control group without any additional stimulation, the results in Figure 3 revealed that releasing cytokines IL-1*β* and IL-1ra had no statistically significant difference in the GL13K-coated titanium group by M1 and M2 macrophages, respectively, but a significant difference can be seen that M1 macrophages seeded on the GL13K-coated surface released a lower level of proinflammatory cytokine TNF-*α*, and M2 macrophages released a higher level of IL-10 than those in the control group. While comparing with the macrophages in the titanium group, M1 and M2 macrophages seeded on the GL13K-coated surface also shows the decreasing expression level of cytokine TNF-*α* and increasing level of cytokine IL10, respectively. This result suggested that GL13K-coated titanium not only regulates macrophage polarization but also regulates the secretion of inflammatory cytokines by different types of macrophage.

### 3.4. Inflammatory and Anti-Inflammatory Gene Expression

**Figure 4:**
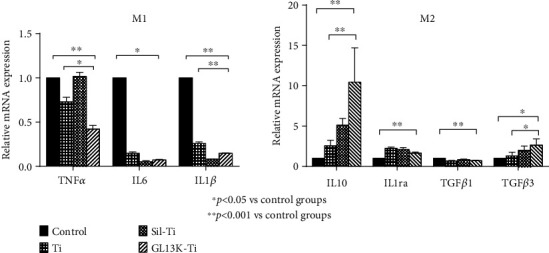
Quantitative analysis of the secretion of TNF-*α*, IL-6, and IL-1*β* of macrophages with M1 polarization and IL-10, IL-1ra, TGF-*β*1, and TGF-*β*3 of macrophages with M2 polarization after culturing on the titanium surface, the silanized titanium surface, and the GL13K-coated surface for 3 days. Error bars represent mean ± SD for *n* = 5. A downregulation of the relative mRNA expression of TNF-*α* (*p* = 0.001) and IL-1*β* (*p* < 0.001) was detected in the groups of GL13K immobilized titanium, while the expression of IL-10 (*p* < 0.001) and TGF-*β*3 (*p* = 0.003) levels were upregulated in these groups.

The mRNA expression of the cytokines IL-1*β* TNF-*α*, and IL-6 in macrophages with M1 polarization and the cytokines IL-10, IL-1ra, TGF-*β*1, and TGF-*β*3 in macrophages with M2 polarization which were cultured on different samples was investigated by qRT-PCR at 72 h.  Figure 4 shows the downregulation of proinflammatory genes TNF-*α* and IL-6 in M1 macrophages after being seeded on the GL13K-coated Ti surface. Whereas this surface also significantly improved the expression level of anti-inflammatory genes IL-10 and TGF-*β*3 in M2 macrophages after being seeded on the GL13K-coated Ti surface. This result also proved that GL13K-coated titanium surface has a property of regulating the cytokine release by macrophages, and it can be seen that this specified surface performed better than titanium surface.

## 4. Discussion

Macrophages play multiple significant roles in the process of osteogenesis and osseointegration after implantation. In the local environment of injured tissue, macrophages first exhibit M1 phenotypes, secreting a large amount of TNF-*α*, IL-1*β*, and IL-6, which can activate inflammatory responses and the tissue regeneration process. However, if these cytokines exist for a long time in healing tissue, they inhibit the expression of BMP-2 receptors and affect the chemotaxis and migration of osteoblasts, thereby affecting the bone formation [18]. In contrast, moderate M2 polarization promotes osteogenesis, while excessive M2 macrophage polarization induces fibrocysts that prevent the inflammatory sites from bone formation. Therefore, the moderate transformation of macrophages from the proinflammatory phenotype M1 to the immune regulation or anti-inflammatory phenotype M2 is considered an important aspect to promote the bone healing process, that is, to promote the functional recovery rather than the formation of scar tissue [19–24].

Our study shows that when the macrophages were cultured on GL13K-coated titanium surfaces, the surface markers of M1 polarization macrophage were reduced and M2 macrophage polarization surface markers were increased compared with those cultured on the titanium surface, which means macrophages can be promoted from the M1 polarization state toward the M2 polarization state while stimulated by GL13K-coated titanium surface. In the cell proliferation experiment of M1 and M2 macrophages, GL13K-coated titanium surfaces were found to inhibit the M1 macrophage polarization and have a good biocompatibility for the polarization of the M2 macrophages. It can be suggested that the regulation of the polarization state of macrophages on the surface of GL13K modified titanium material is due to its different proliferation promotion and inhibition effects on macrophages with different polarization phenotypes. Thanks to the moderation of the promotion effect in M2 macrophages, the GL13K-coated titanium surface can modulate the switch pattern of macrophages by transforming the M1 polarization to M2 polarization mildly without resulting in excessive M2 polarization [19].

In this study, it was also found that the immunoregulatory function of GL13K-coated titanium surface for the expression of anti-inflammatory and proinflammatory factors in macrophages not only derives from its regulation on the transformation of M1/M2 polarized phenotype in macrophages but also from its regulation on the secretion of cytokines by macrophages with different polarized phenotypes. In comparison to M1 macrophages cultured on the titanium surface, those cultured on the surface of GL13K-coated titanium have decreased levels of the inflammatory cytokine TNF-*α*, which has been observed to suppress the differentiation of osteoblastic cells by inhibiting the release of BMP-2 and inducing apoptosis effects on osteoblasts [25, 26]. On the other hand, M2 macrophages released more IL-10 after culturing on the GL13K-coated titanium surfaces. Moreover, compared with the titanium surface, after being cultured on the GL13K immobilized titanium surface for 3 days, the expression levels of mRNA TNF-*α* and IL-1*β* of M1 macrophages decreased, while the mRNA IL10 and TGF-*β*3 expressed by M2 macrophages significantly increased. The inhibitory effect of GL13K-coated titanium surface to the M1 macrophages may be one of the reasons for this, but it also shows that the GL13K immobilized titanium surface can regulate the release of cytokines from macrophages. It can inhibit the proinflammatory factor secreting by M1 macrophages and promote the anti-inflammatory factor releasing of M2 macrophages, thus promoting the transformation of inflammatory processes to the tissue healing process. This result is corresponding with some other researchers that suggested the properties of the antimicrobial peptides to suppress the secretion of inflammation-related cytokines [27].

It is worth noting that the silanized titanium surface also shows some potential of immune regulation in this study. After culturing on this surface for 72 h, M2 macrophage proliferation is promoted (*p* < 0.001), and the silanized titanium surface also showed inhibition in the expression of the cytokine TNF-*α* and mRNA IL-1*β*. As a chemical linker between the biomaterial substrate and the biomolecules, the silanes have been thoroughly studied. Based on silanes, bioactive molecules can covalently attach to surfaces with self-assembled monolayers [28], and we can improve the biological properties of the biomaterial coating using silanes that induce specific cell responses, such as cell proliferation, cell differentiation [29], or antibacterial effects [30, 31]. However, research on the biological properties of these silanized surfaces remains scarce. Previous studies have shown that silanization can improve the surface properties of titanium [32], improve its antibacterial properties [33], and even increase the expression of osteoblastic cell differentiation markers to provide osteoinductive properties [34]. This indicates that it had certain regulation effects on the biological behavior of cells, suggesting that part of the biological function of the GL13K-modified titanium surface was derived from silanization treatment.

In summary, the GL13K immobilized titanium surface was showed the properties for the regulation of macrophages' polarization and the expression of inflammatory and anti-inflammatory effects within the limits of our research. However, further researches are still needed to investigate the mechanism for the biomaterials to regulate the immune responses and induce bone regeneration. In recent years, miRNAs have been demonstrated to be pivotal players actively participating in the macrophage polarization, inflammatory, anti-inflammatory, and tissue healing activities [35]. Moreover, specific miRNA expression profiles have been found to predict specific clinical outcomes, which means miRNAs may be reliable markers and important regulatory elements in the interaction between biomaterials and tissues [36, 37]. This may provide a favorable complement to our future research. It is also worth noting that as every in vitro study, the translation of the results from these studies to the clinical situation is limited. Further researches are still needed to accurately simulate the complex in vivo environment by investigating the involvement of more immunocytes and bone cells.

## 5. Conclusion

In the present study, the GL13K immobilized titanium surface showed the inhibition of the M1 macrophage polarization and a good biocompatibility for the polarization of the M2 macrophages, and it regulates the properties of the inflammatory and anti-inflammatory cytokines, respectively, secreted by M1 and M2 macrophages. Moreover, compared with the titanium surface, the GL13K immobilized titanium surface can regulate the expression of proinflammatory and anti-inflammatory relative genes in M1 and M2 macrophages, resulting in less time in the inflammatory process and more time in the tissue healing process.

## Figures and Tables

**Table 1:** Inflammatory and anti-inflammatory gene primer sequences used in the qRT-PCR.

GAPDH	Forward primer	5′-CTCCCACTCTTCCACCTTCG-3′
	Reverse primer	5′-TTGCTGTAGCCGTATTCATT-3′
TNF-*α*	Forward primer	5′-CTGAACTTCGGGGTGATCGG-3′
	Reverse primer	5′-GGCTTGTCACTCGAATTTTGAGA-3′
IL-1*β*	Forward primer	5′-TGGAGAGTGTGGATCCCAAG-3′
	Reverse primer	5′-GGTGCTGATGTACCAGTTGG-3′
IL-6	Forward primer	5′-ATAGTCCTTCCTACCCCAATTTCC-3′
	Reverse primer	5′-GATGAATTGGATGGTCTTGGTCC-3′
IL-10	Forward primer	5′-GAGAAGCATGGCCCAGAAATC-3′
	Reverse primer	5′-GAGAAATCGATGACAGCGCC-3′
IL-1ra	Forward primer	5′-CTCCAGCTGGAGGAAGTTAAC-3′
	Reverse primer	5′-CTGACTCAAAGCTGGTGGTG-3′
TGF-*β*1	Forward primer	5′-CAGTACAGCAAGGTCCTTGC-3′
	Reverse primer	5′-ACGTAGTAGACGATGGGCAG-3′
TGF-*β*3	Forward primer	5′-CAACACCCTGAACCCAGAG-3′
	Reverse primer	5′-CTTCACCACCATGTTGGACAG-3′

## Data Availability

All data used during this study are available from the corresponding author by request.

## References

[1] Wu C., Chen Z., Yi D., Chang J., Xiao Y. (2014). Multidirectional effects of Sr-, Mg-, and Si-containing bioceramic coatings with high bonding strength on inflammation, osteoclastogenesis, and osteogenesis. *ACS Applied Materials & Interfaces*.

[2] Chen Z., Yi D., Zheng X., Chang J., Wu C., Xiao Y. (2014). Nutrient element-based bioceramic coatings on titanium alloy stimulating osteogenesis by inducing beneficial osteoimmmunomodulation. *Journal of Materials Chemistry B*.

[3] Shi M., Chen Z., Farnaghi S. (2016). Copper-doped mesoporous silica nanospheres, a promising immunomodulatory agent for inducing osteogenesis. *Acta Biomaterialia*.

[4] Zhang W., Zhao F., Huang D., Fu X., Li X., Chen X. (2016). Strontium-substituted submicrometer bioactive glasses modulate macrophage responses for improved bone regeneration. *ACS Applied Materials & Interfaces*.

[5] Chen Z., Klein T., Murray R. Z. (2016). Osteoimmunomodulation for the development of advanced bone biomaterials. *Materials Today*.

[6] Italiani P., Boraschi D. (2014). From monocytes to M1/M2 macrophages: phenotypical vs. functional Differentiation. *Frontiers in Immunology*.

[7] Mills C. D., Kincaid K., Alt J. M., Heilman M. J., Hill A. M. (2017). Pillars Article: M-1/M-2 Macrophages and the Th1/Th2 Paradigm. J. Immunol. 2000. 164: 6166–6173. *The Journal of Immunology*.

[8] Gordon S., Taylor P. R. (2005). Monocyte and macrophage heterogeneity. *Nature Reviews Immunology*.

[9] Sica A., Mantovani A. (2012). Macrophage plasticity and polarization: in vivo veritas. *The Journal of Clinical Investigation*.

[10] Guihard P., Danger Y., Brounais B. (2012). Induction of osteogenesis in mesenchymal stem cells by activated monocytes/macrophages depends on oncostatin M signaling. *Bone*.

[11] Freytes D. O., Kang J. W., Marcos-Campos I., Vunjak-Novakovic G. (2013). Macrophages modulate the viability and growth of human mesenchymal stem cells. *Journal of Cellular Biochemistry*.

[12] Chen Z., Yuen J., Crawford R., Chang J., Wu C., Xiao Y. (2015). The effect of osteoimmunomodulation on the osteogenic effects of cobalt incorporated *β*-tricalcium phosphate. *Biomaterials*.

[13] Kazemzadeh-Narbat M., Kindrachuk J., Duan K., Jenssen H., Hancock R. E. W., Wang R. (2010). Antimicrobial peptides on calcium phosphate-coated titanium for the prevention of implant-associated infections. *Biomaterials*.

[14] Warnke P. H., Voss E., Russo P. A. J. (2013). Antimicrobial peptide coating of dental implants: biocompatibility assessment of recombinant human beta defensin-2 for human cells. *The International Journal of Oral & Maxillofacial Implants*.

[15] Zhou L., Lai Y., Huang W. (2015). Biofunctionalization of microgroove titanium surfaces with an antimicrobial peptide to enhance their bactericidal activity and cytocompatibility. *Colloids and Surfaces B: Biointerfaces*.

[16] Holmberg K. V., Abdolhosseini M., Li Y., Chen X., Gorr S. U., Aparicio C. (2013). Bio-inspired stable antimicrobial peptide coatings for dental applications. *Acta Biomaterialia*.

[17] Zhou L., Lin Z., Ding J., Huang W., Chen J., Wu D. (2017). Inflammatory and biocompatibility evaluation of antimicrobial peptide GL13K immobilized onto titanium by silanization. *Colloids and Surfaces B: Biointerfaces*.

[18] Hengartner N. E., Fiedler J., Ignatius A., Brenner R. E. (2013). IL-1*β* inhibits human osteoblast migration. *Molecular Medicine*.

[19] Brown B. N., Londono R., Tottey S. (2012). Macrophage phenotype as a predictor of constructive remodeling following the implantation of biologically derived surgical mesh materials. *Acta Biomaterialia*.

[20] Sussman E. M., Halpin M. C., Muster J., Moon R. T., Ratner B. D. (2014). Porous implants modulate healing and induce shifts in local macrophage polarization in the foreign body reaction. *Annals of Biomedical Engineering*.

[21] Guo R., Merkel A. R., Sterling J. A., Davidson J. M., Guelcher S. A. (2015). Substrate modulus of 3D-printed scaffolds regulates the regenerative response in subcutaneous implants through the macrophage phenotype and Wnt signaling. *Biomaterials*.

[22] Fearing B. V., Van Dyke M. E. (2014). In vitro response of macrophage polarization to a keratin biomaterial. *Acta Biomaterialia*.

[23] Brown B. N., Valentin J. E., Stewart-Akers A. M., McCabe G. P., Badylak S. F. (2009). Macrophage phenotype and remodeling outcomes in response to biologic scaffolds with and without a cellular component. *Biomaterials*.

[24] Madden L. R., Mortisen D. J., Sussman E. M. (2010). Proangiogenic scaffolds as functional templates for cardiac tissue engineering. *Proceedings of the National Academy of Sciences of the United States of America*.

[25] Gilbert L., He X., Farmer P. (2000). Inhibition of osteoblast differentiation by tumor necrosis factor-*α*^∗^. *Endocrinology*.

[26] Feldmann M., Maini R. N. (2010). Anti-TNF therapy, from rationale to standard of care: what lessons has it taught us?. *Journal of Immunology*.

[27] Liu Y., Xia X., Xu L., Wang Y. Z. (2013). Design of hybrid *β*-hairpin peptides with enhanced cell specificity and potent anti-inflammatory activity. *Biomaterials*.

[28] Bauer S., Schmuki P., von der Mark K., Park J. (2013). Engineering biocompatible implant surfaces. *Progress in Materials Science*.

[29] Toworfe G. K., Bhattacharyya S., Composto R. J., Adams C. S., Shapiro I. M., Ducheyne P. (2009). Effect of functional end groups of silane self-assembled monolayer surfaces on apatite formation, fibronectin adsorption and osteoblast cell function. *Journal of Tissue Engineering and Regenerative Medicine*.

[30] Ma Y., Chen M., Jones J. E., Ritts A. C., Yu Q., Sun H. (2012). Inhibition of Staphylococcus epidermidis biofilm by trimethylsilane plasma coating. *Antimicrobial Agents and Chemotherapy*.

[31] Katsikogianni M. G., Missirlis Y. F. (2010). Interactions of bacteria with specific biomaterial surface chemistries under flow conditions. *Acta Biomaterialia*.

[32] Matinlinna J. P., Tsoi J. K. H., de Vries J., Busscher H. J. (2013). Characterization of novel silane coatings on titanium implant surfaces. *Clinical Oral Implants Research*.

[33] Villard N., Seneviratne C., Tsoi J. K. H., Heinonen M., Matinlinna J. (2015). Candida albicans aspects of novel silane system-coated titanium and zirconia implant surfaces. *Clinical Oral Implants Research*.

[34] Godoy-Gallardo M., Guillem-Marti J., Sevilla P., Manero J. M., Gil F. J., Rodriguez D. (2016). Anhydride-functional silane immobilized onto titanium surfaces induces osteoblast cell differentiation and reduces bacterial adhesion and biofilm formation. *Materials Science and Engineering: C*.

[35] Curtale G., Rubino M., Locati M. (2019). MicroRNAs as molecular switches in macrophage activation. *Frontiers in Immunology*.

[36] Menini M., Pesce P., Baldi D., Coronel Vargas G., Pera P., Izzotti A. (2019). Prediction of titanium implant success by analysis of microRNA expression in peri-implant tissue. A 5-year follow-up study. *Journal of Clinical Medicine*.

[37] Menini M., Dellepiane E., Baldi D., Longobardi M. G., Pera P., Izzotti A. (2017). Microarray expression in peri-implant tissue next to different titanium implant surfaces predicts clinical outcomes: a split-mouth study. *Clinical Oral Implants Research*.

